# Ex vivo gene therapy using intravitreal injection of GDNF-secreting mouse embryonic stem cells in a rat model of retinal degeneration

**Published:** 2009-05-13

**Authors:** Kevin Gregory-Evans, Francis Chang, Matthew D. Hodges, Cheryl Y. Gregory-Evans

**Affiliations:** 1 Department of Clinical Neuroscience, Faculty of Medicine, Imperial College London, London, UK; 2 Western Eye Hospital, Imperial College Healthcare NHS Trust, London, UK

## Abstract

**Purpose:**

Safe and prolonged drug delivery to the retina is a key obstacle to overcome in the development of new medicines aimed at treating progressive retinal disease. We took advantage of the ability of embryonic stem cells to survive long-term in foreign tissue and used these cells to deliver neuroprotectant molecules to the retina of the rhodopsin TgN S334ter-4 rat model of retinitis pigmentosa (RP).

**Methods:**

Mouse embryonic stem (mES) cells, derived from the pluripotent embryonic stem cell line E14TG2a, were genetically engineered to oversecrete the glial cell-derived neurotrophic factor (GDNF). Cell suspensions, containing approximately 200,000 cells and expressing approximately 35ng/10^6^ cells/24 h GDNF, were injected into the vitreous cavity of TgN S334ter rat eyes at postnatal day 21 (P21) without immunosuppression. Histological and immunofluorescence imaging was used to evaluate photoreceptor survival up to P90. Local (vitreous) and systemic (serum) concentrations of GDNF were determined and ocular side effects were monitored.

**Results:**

Green fluorescent protein (GFP)-expressing mES cells were observed on the inner limiting membrane of the retina in retinal flatmounts up to P90. In cryostat sections at P45, some GFP-expressing cells had integrated into the inner retina, but did not migrate into the outer nuclear layer. After an initial lag period, the photoreceptor cell counts were significantly higher (p≤0.05) in animals treated with GDNF-secreting mES cells than in untreated animals, principally in the peripheral retina. Several adverse side effects such as tractional detachments and areas of hyperplasia were seen in a minimal number of treated eyes. Abnormally high levels of GDNF in the peripheral circulation were also observed.

**Conclusions:**

ES cells engineered to secrete GDNF exerted a neuroprotective effect for at least three months on retinal structure in the TgN S334ter rat model of retinal degeneration. Immunosuppression was not required for this. Several adverse effects were identified which require further investigation to make cell-based delivery of neuroprotection a viable clinical strategy.

## Introduction

Apoptotic cell death is a central process in the pathophysiology of a diverse number of diseases of the central nervous system (CNS). In the eye, apoptotic cell death is a key factor in many blinding retinal diseases, such as retinitis pigmentosa (RP) [1], and also the atrophic (dry) form of age-related macular degeneration [2]. Such retinal diseases are among the most common causes of blindness in the developed world [3]. Any therapeutic process that can impede retinal cell death could therefore have a major impact on the prevalence of blindness. Over the last decade the concept of neuroprotection, particularly aimed at the CNS, has emerged with the goal of inhibiting apoptotic cell death through pharmacological means [4-6].

Pioneering work by Faktorovich and coworkers revealed delayed photoreceptor degeneration in the Royal College of Surgeons rat using basic fibroblast growth factor (bFGF) [7]. Since then, considerable evidence has emerged to show that a range of neurotrophic (survival) growth factors can inhibit retinal degeneration in several animal models [8,9]. These include glial-derived neurotrophic factor (GDNF) [10,11], brain-derived neurotrophic factor (BDNF) [12], ciliary neurotrophic factor (CNTF) [12,13], lens epithelium-derived growth factor (LEDGF) [14], pigment epithelium-derived factor (PEDF) [15], and rod-derived cone viability factor (RdCVF) [16]. Clinical studies to date however have reported mixed results. Significant progress with CNTF use in outer retinal degeneration patients has been reported [17,18], but less success has been seen using memantine (an N-methyl D-aspartate-type [NMDA-type] glutamatergic channel blocker with neuroprotective action [19]) in patients with ganglion cell disease [20].

Most neurotrophic agents that have been studied in retinal disease are either too large to cross the blood–retinal barrier (BRB) [21] or are associated with unacceptable systemic side effects. Thus direct delivery to the eye is regarded as the only practical way to deliver neuroprotection. This would however necessitate repeated and frequent intraocular injections. Several long-term delivery methods have been proposed to manage this problem. These include: slow release capsules [13,22]; coating onto beads [10,23]; transfecting retinal cells in situ to secrete neuroprotectant [24]; and trans-scleral delivery of drug [25,26]. In addition cell-based delivery of neuroprotectant has also been investigated [27-29], although the best cell to use has yet to be established. Therefore, in this study we undertook preliminary, “proof-of-principle” work to determine whether genetically modified embryonic stem cells, injected into the vitreous cavity, could be useful in the long-term delivery of neuroprotectant in retinal degeneration. In particular we specifically assessed the likely limitations of this technology.

## Methods

### Engineering of mammalian expression vector, pGDNF.GFP

We engineered a plasmid (that we named ‘pGDNF.GFP’) through several cloning steps to constitutively express GFP and GDNF. The open reading frame of human GDNF (AY893733; obtained from human cDNA sequence; Dana-Farber/Harvard Cancer Centre DNA Resource) was amplified by PCR with a reverse primer containing a stop codon. In addition the primers contained XbaI and PacI linkers to facilitate directional cloning. This PCR product was digested with XbaI and PacI and subcloned into the polylinker of the mammalian expression vector, pCAβ-linker1 internal ribosome entry site (IRES).GFP (a gift from Caroline J. Formstone, King’s College London, UK). Expression of GDNF is driven by the chicken β-actin promoter under the influence of the cytomegalovirus enhancer [30], and expression of GFP is from the IRES translation initiation sequence. A puromycin resistance gene cassette was also amplified by PCR from a 3′HPRT targeting vector [31] and cloned into the SapI site of the final construct, pCAbeta linker 1 IRES.GFP/hGDNF puro (abbreviated to pGDNF.GFP). Cloned regions were sequenced to ensure plasmid integrity.

### Mouse embryonic stem cell culture

We elected to transfect the expression vector into mouse embryonic stem (mES) cells because of the lack of availability of sufficiently characterized rat ES cells for ex vivo gene therapy. E14TG2a cells (a gift from Dr. Andrew Smith, University of Edinburgh, UK) originally derived from 129 strain/Ola mice [32] were cultured in the absence of a fibroblast feeder layer on 0.1% w/v gelatinized 6 cm plates in complete media (knockout Dulbecco's modified eagle medium [DMEM]; Invitrogen Ltd., Paisley, UK), supplemented with 15% fetal calf serum (Cambrex, Verviers, Belgium), 1% penicillin and streptomycin, 20 mM L-glutamine, 0.1 mM β-mercaptoethanol, and 50 U leukemia inhibitory factor (LIF; Chemicon Europe Ltd, Chandlers Ford, UK). Cultures were maintained at 37 °C with 5% CO_2_ with media replacement every 24 h.

### Stable transfections of GDNF secreting mES cells

The pGDNF.GFP construct was transfected into mES cells by electroporation. Next, mES cells were harvested, then washed in PBS, which contained 137 mM NaCl, 2.7 mM KCl, 4.3 mM Na_2_HPO_4_, and 1.47 mM KH_2_PO_4_, pH of 7.4. Cells were resuspended in PBS at a concentration of 2.5×10^7^ cells in 600 μl. The cell suspension was mixed with 50 μg of SalI linearized pGDNF.GFP plasmid DNA, placed in 0.4 cm electroporation cuvettes, and electroporated in a gene pulser (Bio-Rad, Hemel Hempstead, UK) at 0.8 Kv, 3 μF for 0.2 msec. Cells were resuspended in complete media and plated at a density of 2.5×10^6^/10 cm dish. After 24 h the media was replaced with complete media containing puromycin at 0.4 μg/ml and cells remained in selection for 15 days when colonies became visible. Resistant ES cell colonies (192 in total) were placed into 96 well plates and expanded for five days. Each colony was then replica plated (1:4), and a week later, were assessed for GDNF secretion by Enzyme-linked immunosorbent assay (ELISA) of supernatant.

### Immunocytochemistry

Cells were fixed in 4% PFA (paraformaldehyde) and then, permeabilised in 0.2% Triton X-100/PBS (PBS composition: 137 mM NaCl; 2.7 mM KCl; 4.3 mM Na_2_HPO_4_; 1.47 mM KH_2_PO_4_; pH of 7.4), blocked for 2 h at room temperature in 2% normal serum/0.5% BSA (blocking buffer) and then incubated with primary antibody for 16 h at 4 °C in blocking buffer [33]. Rabbit anti-GFP (Invitrogen) was used to check gene expression in transfected cells and rabbit anti-human OCT4 (Sigma-Aldrich Company Ltd., Gillingham, UK) was used to confirm pluripotency. After washing 3 times in blocking buffer, cells were then incubated with a 1:1000 dilution of a secondary antibody in blocking buffer, conjugated to alkaline phosphatase (goat anti-rabbit IgG) for 30 min at room temperature. Cells were washed three times in PBS-Tween-20 and then developed with 4-Chloro-2-methylbenzenediazonium/3-Hydroxy-2-naphthoic acid 2,4-dimethylanilide phosphate tablets (Fast Red TR/AS-MX Napthol phosphate; Sigma-Aldrich). Cells were mounted with Hydromount and visualized under light microscopy. For immunocytochemistry in tissue sections, GFP expression was examined using a GFP-fluorescein isothiocyanate (FITC) antibody (Abcam plc, Cambridge, UK) and epifluorescence microscopy.

### Disease model

The primary aim of the study was to determine the effectiveness and safety of stem cell delivery of neuroprotectant (in this case GDNF) in an in vivo model. We therefore undertook studies in a model where GDNF had already been shown to have a reproducible neuroprotective effect. Only limited studies of GDNF effectiveness have been undertaken in mouse models of retinal degeneration: *rd1* [10,34] and *Periph2* [11]. We did not consider either of these models to be optimal for our study. One study of GDNF-releasing microspheres showed only brief and limited effectiveness in the *rd1* mouse [10], and another study of subretinal injection of GDNF only resulted in benefit in 4 of 10 *rd1* retinas [34]. In addition, GDNF expression in *Periph2* mice had little effect unless combined with gene replacement therapy [11].

Conversely, GDNF has been shown to effectively neuroprotect in the S334ter rat well beyond the postnatal day 21 (P21) time point. TgN S334ter were donated by Professor Matt LaVail (University of California at San Francisco, San Francisco, CA) [35]. During the study, all animals were housed in standard cages and given food and water ad libidum while being maintained on a 12-h light/dark schedule. All work was performed with adherence to the ARVO Statement for the Use of Animals in Ophthalmic and Vision Research and local Ethical Committee regulations. GDNF has been shown to be an effective neuroprotectant in this model [36]. The insertion of a premature termination codon [37] leads to the translation of a truncated protein lacking the last 15 amino acids, similar to abnormalities of the C-terminal domain found in many human cases of RP [38].

### Cell injections

Expanded E14TG2a mES cells were resuspended in PBS to give a final concentration of 50,000 cells/μl. A Hamilton syringe was used to inject 4 μl of the cell suspension (2×10^5^ cells) into the vitreous cavity of one eye, through an entry site 1–2 mm behind the limbus of P21 TgN S334ter rats. In total three groups of animals were studied. One group of animals was treated with GDNF secreting mES cells (n=34), one group with unmodified mES (n=15), and a final group received sham injections of PBS (n=19). No animals underwent immunosuppression.

### Histological assessment of rat retina treated with GDNF-mES cells

Animals were euthanized by CO_2_ asphyxiation. Eyes were enucleated and marked at the 12 o’clock position, using a black nylon stitch for orientation. For cryostat sectioning, eyes were immersed in 4% PFA for 1 h, cryopreserved in 30% sucrose overnight, snap frozen and then embedded in OCT compound. Either 5 μm sagittal or coronal sections were stained with GFP-FITC antibody and DAPI. The sections were examined with fluorescence microscopy, and montages were made in Photoshop 4.0. Eyes for retinal flatmounts were immersed for 1 h in 4% PFA. The anterior segment was then removed and the eyecup immersed for a further 1 h. The orientation mark was transferred to the retina by cutting a notch and then radial cuts were made toward the optic nerve using sharp scissors. The retina was gently teased off the eye cup as a single piece of tissue, and transferred to a microscope slide, where it was positioned. Several drops of Hydromount were placed on the tissue and a coverslip added. GFP-expressing mES cells were visualized by confocal microscopy. Eyes that would be used for counting photoreceptor nuclei were immersed in half strength Karnovsky's fixative for 1 h, and the anterior segment removed by excision at the ora serata. Posterior segments were embedded in paraffin, cut sagitally to a thickness of 5 μm, and counterstained with hematoxylin and eosin.

### Photoreceptor cell counting

To standardize photoreceptor nuclei counting from different eyes, we only employed sagittal sections that included the optic nerve. Images were overlaid with a 300 μm-length rectangular template using Canvas (ACD Systems, Miami, FL) software. This template was positioned at four predetermined coordinates to give counts from two central (behind the equator) and two peripheral (in front of the equator) coordinates [39]. These four images for each eye were then imported into Photoshop, and photoreceptor nuclei in the outer nuclear layer were labeled with overlaying spots. An exported JPEG of this layer of spots was then counted in ImageJ v 1.37.software to give the total number of outer nuclear layer nuclei in each grid.

### ELISA assays

The concentration of GDNF protein in tissue culture supernatant, rat serum, and rat vitreous was determined with the GDNF Emax^®^ ImmunoAssay System (Promega Ltd., Southampton, UK), which was used according to manufacturer’s instructions. Briefly, a 96 well plate was coated with anti-GDNF monoclonal antibody. The samples were then applied to the plate overnight, washed three times with wash buffer (20mM Tris-HCl pH 7.6; 150mM NaCl; 0.05% (v/v) Tween® 20), and incubated with a polyclonal antibody to GDNF. Samples were incubated with a secondary anti-chicken immunoglobulin Y antibody conjugated to horseradish peroxidase and detected using a chromogenic substrate. The color change was measured at 450 nm using a spectrophotometer.

### Statistical analysis

Data was collected from eyes treated with intravitreal injections of either GDNF-secreting mES cells, unmodified mES cells, or vehicle only. Results were compared as mean±standard error of the mean (SEM). Nuclei counts were compared between groups using the non-parametric Mann–Whitney test (Minitab statistical software pack version 15.0) to account for comparison between groups of different size. Statistical significance was set as p≤0.05. For GDNF levels in serum and vitreous samples a paired Student *t*-test was used. Data from each time point in control mES cell-injected groups was compared to data from GDNF-mES cell-treated groups and analyzed for statistical significance.

## Results

### GDNF-secreting mES cell line production

**Figure 1:**
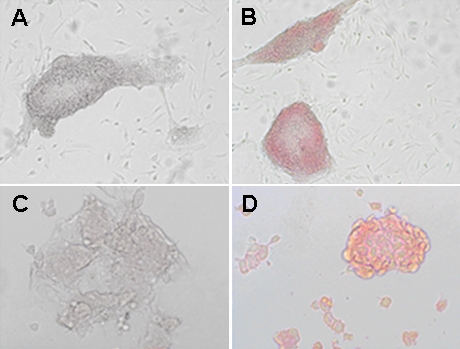
GFP and Oct4 expression in mES cells transfected with pGDNF. Cells expressing this GDNF plasmid also express fluorescent protein GFP and will appear red colored when exposed to GFP antibody. **A:** Control experiment, no red staining if mES cells are not exposed to anti-GFP antibody. **B**: Immunostaining of mES cells in culture using an anti-GFP antibody gives cells a red coloration confirming GFP cell expression. **C:** Control experiment, no red staining if mES cells are not exposed to anti- OCT4 antibody. **D**: Cells immunostained using an anti-human OCT4 (POU5F1) antibody appear red, confirming Oct4 cell expression and establishing cell totipotency.

Several rounds of transfection with pGDNF.GFP were performed with increasing concentrations of puromycin as a selectable marker for integration of the plasmid. Presence of GFP expression in mES cells in culture compared to controls was confirmed by immunocytochemistry (Figure 1A,B). mES cell clones were also screened for GDNF secretion into supernatant by ELISA. GDNF was quantified using regression analysis based on the standard curve plotted from analysis of known concentrations of a GDNF standard. In the first attempt, nine colonies (0.4 μg/ml puromycin) were generated ranging in GDNF secretion of 6.45–10.2 ng/GDNF/10^6^ cells/24 h. Subsequently increasing puromycin selection to 2 μg/ml generated four clones with GDNF secretion into supernatant ranging from 11.1 to 36.0 ng/GDNF/10^6^ cells/24 h.

### Assessment of pluripotency in GDNF-secreting mES cells

The *Oct4* gene encodes a transcription factor that is expressed in mES cells. Oct4 is crucial for the maintenance of embryonic cell pluripotency. Downregulation of Oct4 would indicate cell differentiation with possible loss of the tissue integration characteristics of pluripotent ES cells [40]. Immunocytochemistry in clumps of mES cells, expressing 36 ng GDNF/10^6^ cells/24 h, showed Oct4 expression in all cells (Figure 1C,D). This established that this cell line remained pluripotent in tissue culture.

### mES cell survival within retinal tissue

**Figure 2:**
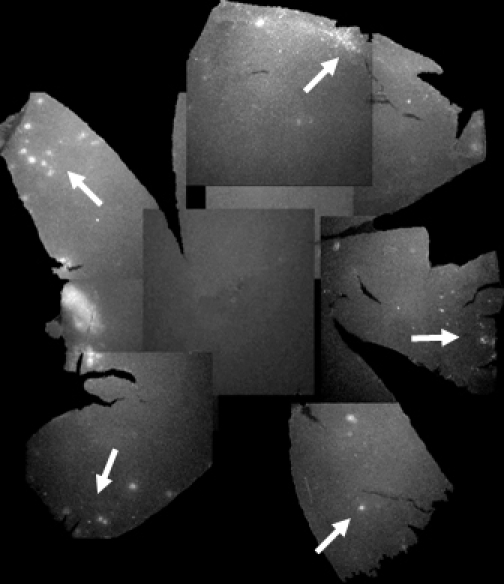
Composite flatmount images from a single TgN S334ter treated retina at P70. One rat eye from the group treated with intravitreal glial-derived neurotrophic factor (GDNF)-secreting mouse embryonic stem (mES) cells at P21. Spots of increased hyperfluorescence (white), indicative of colonies of green fluorescent protein (GFP)-expressing mES cells, are mainly seen in the peripheral retina (arrows).

**Figure 3:**
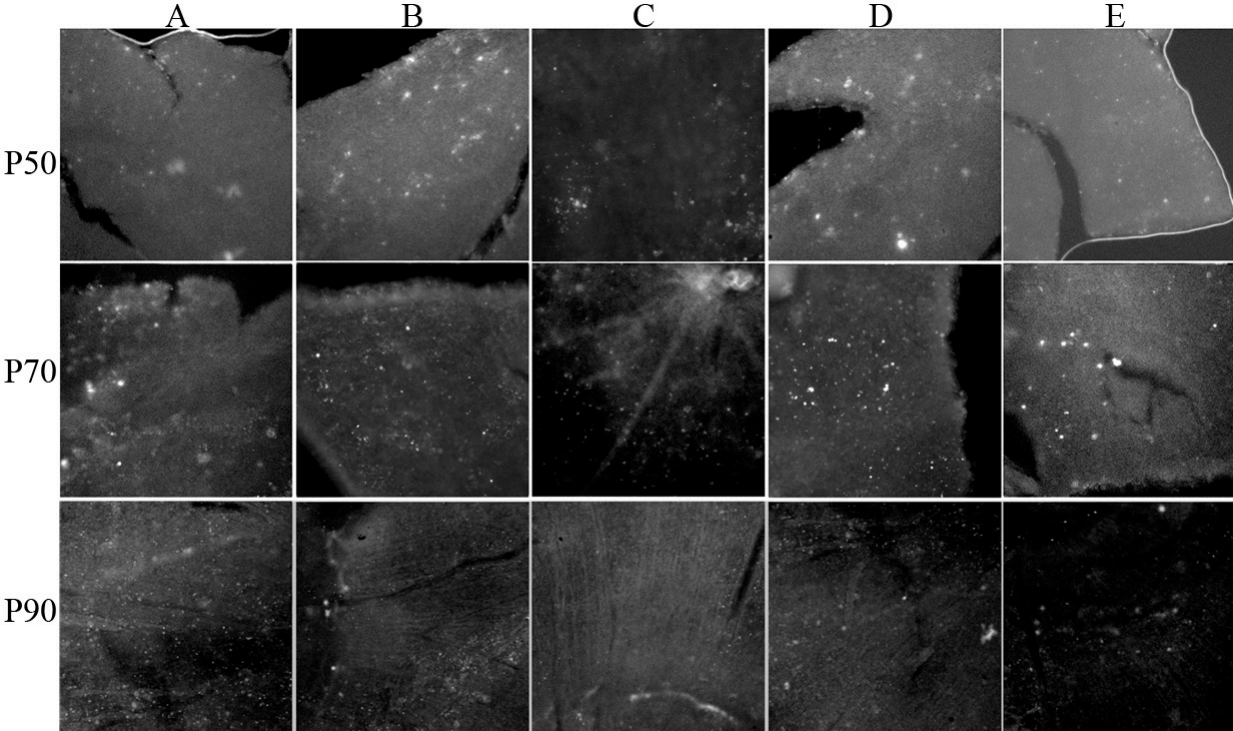
Flatmount images of TgN S334ter rat retina treated with GDNF-secreting mES cells. Rows indicate assessments at time points P50, P70, and P90. Columns, represent images from retina from **A:** upper nasal; **B:** upper temporal; **C:** central; **D:** lower nasal; **E:** lower temporal areas. All rats were from the group treated with intravitreal glial-derived neurotrophic factor (GDNF)-secreting mouse embryonic stem (mES) cells at P21. White spots indicative of colonies of green fluorescent protein (GFP)-expressing mES cells appear mainly on the surface of the peripheral retina rather than central retina. As time progresses, clumps become more diffuse and ill-defined, suggesting either migration into the retinal tissue or cell loss.

Retinal flat mounts from eyes at P50, P70, and P90 were used to observe mES cell survival and cell distribution over time. GFP-expressing cells did not appear to be distributed uniformly across the retinal surface (Figure 2). Interestingly, at all time points studied, the majority of GFP-expressing cells were found to localize to the peripheral retina, although most retinas did have some cells centrally as well. By P90, clumping of autofluorescence was less apparent and a more diffuse fluorescence was evident (Figure 3). This could have been due to cell migration from the surface into the substance of the retina.

### In vivo integration of mES cells into retinal tissue

**Figure 4:**
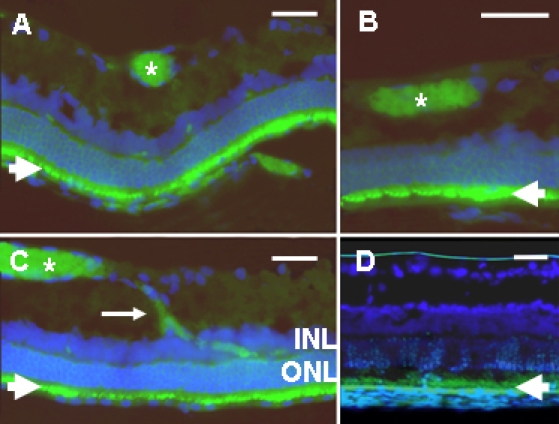
Cryosections of TgN S334ter rat retina studied at post-natal day 35. All sections were immunostained for green fluorescent protein (GFP; green) and retinal cell nuclei were counter-stained blue with DAPI. **A-C:** Illustrates images from rats from the group treated at post-natal day 21 with intravitreal injection of GDNF-secreting (and GFP-expressing) mouse embryonic stem cells (mES cells). Plates **A-C** demonstrates integration of GFP-expressing mES cells into the retina. The asterisks mark clumps of mES cells. Some are seen migrating (arrow in **C**) from the innermost retina toward the outer retina, and extending between the outer nuclear layer (ONL) and inner nuclear layer (INL). **D:** Illustrates a mock injection eye from the group treated with intravitreal injection of sham injection of PBS, and shows autofluorescence in the retinal pigment epithelium and photoreceptor outer segments (arrowhead). Abbreviations: inner nuclear layer (INL); outer nuclear layer (ONL). Scale bars represent 50 μm.

In TgN S334ter eyes treated with GDNF-secreting mES cells, fluorescence imaging of retinal sections identified distinct, elongated, regions of GFP-staining cell clumps adherent to the inner limiting membrane (Figure 4). In several instances the GFP-staining cells could be seen traversing the inner plexiform layer to line the boundary between the inner plexiform layer and the inner nuclear layer (Figure 4C). We did not see integration into the outer nuclear layer, consistent with observations made by others [41-43]. Sections from untreated TgN S334ter eyes or those injected with control mES cells exhibited fluorescence only within the retinal pigmented epithelium or, weakly, within the photoreceptor outer segments (Figure 4D). Due to lipofuscin accumulation autofluorescence is a well known feature of the retinal pigment epithelial cells and is a result of aging or retinal degeneration. Autofluorescence is also often detectable in the outer segments due to the tightly packed membranes (personal communication, John Flannery, University of California, Berkeley, San Francisco, CA).

### Histological rescue

**Figure 5:**
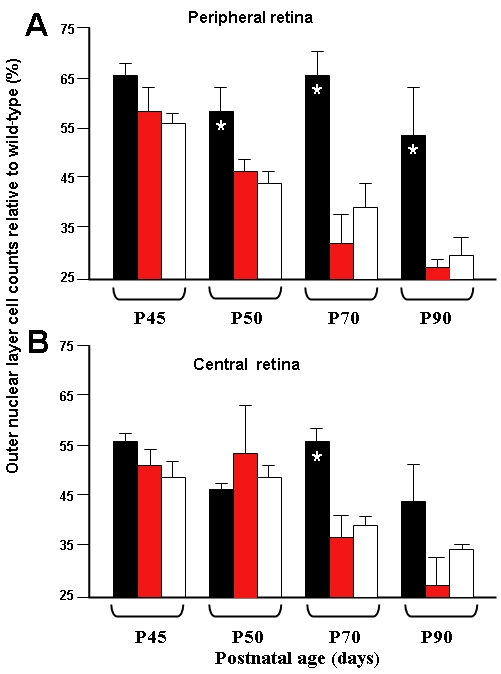
Treated TgN S334ter rat retina photoreceptor cell nuclei counts. Animals received injections at P21 and cell counts were undertaken at periods up to P90. Graph **A** represents cell nuclei counts from peripheral retina. Graph **B** presents cell nuclei counts from posterior pole (central) retina. Cell counts were undertaken in six eyes treated with glial-derived neurotrophic factor (GDNF)-secreting mouse embryonic stem (mES) cells, black bars; in another 8 eyes treated with unmodified mES cells, hatched bars; and in nine eyes treated with sham injections, white bars. Data are presented as the mean percentage of wild-type counts±SEM and analyzed for statistical significance with a Mann–Whitney test. The asterisk indicates p<0.05. The figure demonstrates that preservation of photoreceptor cell nuclei was seen only in eyes treated with GDNF-secreting mES cells and mostly in peripheral retina tissue.

Eyes from the three treatment groups (GDNF-mES cells, unmodified mES cells, sham PBS injection) were sectioned and outer nuclear layer (photoreceptor) cell counts undertaken. When data from all retinal coordinates for all time points were pooled together for GDNF-secreting mES cell-treated retina versus sham controls, a statistically significant difference was seen suggesting a relative preservation of photoreceptors with GDNF-secreting mES cells (p<0.001). However, it was evident that this relative preservation of nuclei was mostly from counts pooled from peripheral coordinates (p<0.001) rather than from counts pooled from central coordinates (p=0.15). At individual time points, statistically significant peripheral retina preservation was seen at P50, P70, and P90 (p<0.05). In comparison for central retina, preservation of cell nuclei was only statistically significant at P70 (p<0.05; Figure 5). No statistically significant difference was seen when data from unmodified mES cell-treated animals was compared with sham-treated controls, suggesting that the preservation of outer nuclear layer cells was not due to an inherent neuroprotective effect of mES cells.

### Local ocular adverse effects

No systemic adverse event was recorded after intravitreal injection. However, several ocular side effects were observed (Table 1). Of 68 injections, one eye (treated with sham injection) had a severe, purulent discharge which was assumed to be an endophthalmitis. Other side effects included lens opacities (8%), total retinal detachment (3%), and minor tractional detachments in 5% of cases. None of these adversely affected eyes were used in cell count experiments. Several eyes exhibited areas of focal thickening of outer nuclear layer of the retina relative to surrounding areas. These were often seen to be over 20 cells thick with sharply defined boarders. This is more than would be expected in age-matched wild-type or TgN S344ter eyes at corresponding retinal coordinates [39,44]. These appeared to represent areas of hyperplasia and did not occur in the inner nuclear or ganglion cell layers.

**Table 1:** Side effects associated with intravitreal injection into TgN S334ter rat eyes.

**Treatment**	**Endophthalmitis**	**Cataract**	**Total retinal detachment**	**Minor traction detachment**
GDNF-secreting mES cells (n=34)	0	2	0	2
Unmodified mES cells (n=15)	0	3	2	1
PBS sham injections (n=19)	1	0	0	0

Data from 68 treated eyes. These data show that complications were seen in rats regardless of the type of intravitreal injection but that retinal side effects were seen only in rats treated with unmodified or GDNF-secreting mES cells. Abbreviations: glial-derived neurotrophic factor (GDNF), mouse embryonic stem (mES), phosphate buffer solution (PBS).

### In vivo measurement of GDNF concentration in ocular tissue and blood

To investigate potential long-term systemic consequences to exposure to elevated levels of GDNF, the concentration of GDNF was determined in blood and vitreous samples from wild-type, untreated, control, and treated animals at three different time points. ELISA results suggested that compared to control eyes (mES cells only), treatment with GDNF-secreting mES cells significantly increased vitreous levels as might be expected after intravitreal injection (range 6–16 fold over the three time points; p<0.01). However, GDNF levels were also significantly increased in serum samples (range 10–12 fold; p<0.001), raising the serious possibility of systemic adverse effects due to long-term exposure to GDNF (Table 2).

**Table 2:** GDNF concentrations in vitreous and serum samples

**Sample time point**	**Wild-Type**		**Untreated S334ter**	
**Serum (pg/ml)**	**Vitreous (pg/ml)**	**Serum** **(pg/ml)**	**Vitreous** **(pg/ml)**
P50	12.8±1.6	21.3±19.4	23.5±3.8	16.5±7.8
P70	16.8±5.2	18.5±6.9	21.4±3.4	19.9±8.7
P90	14.0±4.1	3.8±1.0	28.5±11.0	9.2±10.1
				
	**Control S334ter**		**Treated S334ter**	
	**Serum (pg/ml)**	**Vitreous (pg/ml)**	**Serum (pg/ml)**	**Vitreous (pg/ml)**
P50	29.4±4.9	18.3±7.2	*337.0±8.0	107.8±30.2**
P70	32.6±5.8	11.4±3.7	*333.0±79.8	181.1±32.1**
P90	22.1±7.2	14.2±6.3	*272.0±35.5	222.2±24.0**

Wild-type glial-derived neurotrophic factor (GDNF) levels in the serum and vitreous were compared to TgN S334ter treated (GDNF-secreting mouse embryonic stem cells), TgN S334ter control (mouse embryonic stem cells only), or untreated S334ter rats that were assessed at P50, P70, and P90 (n=3/time point). Data are presented as mean±SEM. The increase in serum (*p<0.001) or vitreous (**p<0.01) GDNF levels in treated animals compared to control mES cell injections is significant.

## Discussion

This study is the first to investigate the feasibility of embryonic stem cells in delivering neuroprotectant to the retina. The results demonstrated that mES cells, which were engineered to secrete GDNF and were delivered by intravitreal injection to the TgN S334ter rat model of retinal degeneration, have a significant effect on prolonging photoreceptor survival compared to the parent cell line or sham injection. This neuroprotective effect of GDNF was evident for at least three months, suggesting that ES cells could potentially be used as a tool to deliver neuroprotectant to the degenerating eye.

GDNF is a member of the transforming growth factor superfamily, first described as a stimulant of survival of dopaminergic neurons in vitro [45]. Cell lines overexpressing GDNF have neuroprotective effects in models of neurodegenerative disorders such as Parkinson disease [46] and Huntington’s disease [47]. Both GDNF and its receptors are synthesized in the retina [48-50], an indication that this growth factor has an innate neurotrophic role in this tissue. Subretinal injection, slow-release devices, adenoviral delivery, or cell-based delivery of GDNF have been shown to delay photoreceptor loss in several different models of retinal degeneration [10,13,27-29,34,36]. The cellular mechanism of GDNF neuroprotection is however unresolved, as it has been proposed that GDNF-induced rescue of mutant photoreceptors is an indirect effect mediated by retinal Müller glial cells [51].

The use of embryonic stem cells to deliver neuroprotectant to the retina is a valid proposition, based upon their remarkable ability to integrate and survive long-term in mature and particularly diseased tissue [52]. In addition, it has been proposed that stem cells have an inherent neuroprotective ability [41,43,53] that could be augmented by genetic modification. Our data did not support this hypothesis in the TgN S334ter retinal degeneration model because unmodified mES cells alone did not seem to afford a significant protective effect. Remarkably, the relative preservation of photoreceptor cells seen with GDNF-secreting mES cells seemed to be regional in that most effect was seen in cell counts from peripheral retina. This appeared to correlate with the distribution of GDNF-secreting mES cells seen in retinal flat-mounts, suggesting that GDNF effectiveness is confined to the locality of secreting cells. This nonuniform incorporation of cells following intravitreal injection has been observed in several other studies [27,28,42,43] and hence could limit the effectiveness of this approach in human trials. In this context, it would be anticipated that clinical effectiveness would be most appreciated when neuroprotection significantly preserved the central (macula containing) region.

It is not clear why mES cells tend to distribute toward the peripheral retina, but this is the first report of this phenomenon having a functional consequence. It could be in response to signaling from a greater mass of degenerating photoreceptors in peripheral retina [54]. It has been suggested that mesenchymal stem cells, for example, home in on damaged tissue in a manner similar to the way leukocytes traffic from the blood to inflammatory sites, using chemokines and adhesion molecules [55]. Another possibility is that this is a physical effect after injection due to centrifugal and centripetal forces generated by eye movement, or it may be secondary to saccadic eye movement-induced fluid currents within the partially liquefied vitreous [56]. The complex shape of the vitreous cavity has been modeled in fluid dynamics studies. When exposed to saccadic eye movements, particles injected into the fluid tend to flow toward the anterior vitreous, just behind the lens regardless of their initial position [57]. Although this model does not fully reproduce the fibrillar structure of the human vitreous, it does suggest that injected cells would tend to accumulate anterior to the equator and hence tend to concentrate in the peripheral retina. This could have significant consequences for the distribution of effect of any therapeutic agent injected into the vitreous.

Many issues need to be addressed before translating retinal neuroprotection into clinical practice. Among these is the key concern of how best to deliver these drugs safely and with long-term neuroprotective effect. In this study we specifically noted the occurrence of adverse effects: tractional retinal detachments, areas of outer nuclear layer hyperplasia, and abnormally high levels of GDNF in the peripheral circulation. Tractional retinal detachment, being a side effect of GDNF per se, seems unlikely since a gene therapy approach to GDNF delivery specifically revealed no such complications [36]. These tractional detachments could be due to surgical technique, although surgical trauma is relatively minor with intravitreal injections. It is more likely to be due to mES cell adhesion onto the vitreoretinal interface. The molecular and cellular mechanisms resulting in this has not been determined, but may induce a secondary inflammation and thus fibrosis. Tractional detachments have not been detected in other cell-based delivery studies that used immunosuppression [27,28]. When GDNF-secreting microspheres were used, areas of retinal traction near microsphere aggregates were detected, but no actual retinal detachment was observed [10]. Although frequently employed in cell transplantation research, immunosuppression was not undertaken in this study for two reasons. First, some studies suggest that immunosuppression may not be needed when using embryonic stem cells [58], although recently the immune privilege of embryonic stem cells has been questioned [59]. Second, the study was designed more as a practical assessment of the technology rather than a proof of principle experiment. Since long-term immunosuppression will not be feasible in clinical applications, it would seem unhelpful to base studies on its use. However, immunoprivileged cells, e.g., those derived from the subjects undergoing treatment, may be a practical approach to employ in overcoming these immune reactions [60].

Another adverse effect we observed was what appeared to be focal areas of hyperplasia, similar to those seen in other studies [10]. Dystrophic retina often appears to exhibit an undulating variation in outer nuclear layer thickness in comparison to the uniform tapering of wild-type retina progressing from the posterior pole to the ora. However, we found this hyperplasia to be more extreme than this. It could represent local areas of enhanced cell preservation, correlating with the “local” effectiveness of GDNF-secreting mES cells. It might also represent areas of proliferation, although other studies have specifically reported no indications of nerve or glial cell mitogenesis induced by GDNF treatment [36]. This observation could have significant consequences for clinical trials and therefore warrants further investigation in larger cohort studies, such as immunocytochemistry studies using markers of retinal mitosis such as cyclin D1 [61].

No studies to date have reported on local or systemic GDNF measurements in retinal degeneration models treated with GDNF. Raised levels of GDNF in the vitreous would be expected with a cell-based delivery approach as the cells would secrete GDNF at the vitreoretinal interface. In addition the high concentrations detected in the blood seem difficult to explain from a cell source in the eye. It is possible that, rather than GDNF leaking from the eye, actual GDNF-secreting mES cells have made their way into the general circulation. It is unclear what the likely consequences of this might be. One study reports weight loss as a significant complication in a human clinical trial of ventricular injection of GDNF in patients with Parkinson disease [62]. Reduced food consumption was also observed in rhesus macaques receiving infusion of GDNF into the putamen [63] and another study reported cerebellar damage [64]. It has been assumed that little would leak into the general circulation because of the blood–retinal barrier, however this barrier is incomplete in retinal dystophies [65]. Conversely, GDNF is thought to be a key molecule in maintaining the blood–retina barrier [66]. Further investigation is needed to study the cause and effect of this adverse phenomenon.

In summary, this study shows that mES cells engineered to deliver GDNF can prolong photoreceptor survival. However, complicating issues that have been observed would need resolution before proceeding to clinical studies. This is still an attractive proposition, however, because of its applicability to retinal disease of diverse etiologies. With the arrival of clinical grade stem cells [67,68], produced on an industrial scale, it is likely that embryonic stem cells, or their derivatives will become an economically viable cell source and therefore suitable for cell-based therapeutics needed to support huge clinical demand.

## References

[1] Travis GH (1998). Mechanisms of cell death in the inherited retinal degenerations.. Am J Hum Genet.

[2] Dunaief JL, Dentchev T, Ying GS, Milam AH (2002). The role of apoptosis in age-related macular degeneration.. Arch Ophthalmol.

[3] Congdon N, O'Colmain B, Klaver CC, Klein R, Muñoz B, Friedman DS, Kempen J, Taylor HR, Mitchell P, Eye Diseases Prevalence Research Group. (2004). Causes and prevalence of visual impairment among adults in the United States.. Arch Ophthalmol.

[4] Kaur C, Ling EA (2008). Antioxidants and neuroprotection in the adult and developing central nervous system.. Curr Med Chem.

[5] Gonzalez FF, Ferriero DM (2008). Therapeutics for neonatal brain injury.. Pharmacol Ther.

[6] Barnstable CJ, Tombran-Tink J (2006). Molecular mechanisms of neuroprotection in the eye.. Adv Exp Med Biol.

[7] Faktorovich EG, Steinberg RH, Yasumura D, Matthes MT, LaVail MM (1990). Photoreceptor degeneration in inherited retinal dystrophy delayed by basic fibroblast growth factor.. Nature.

[8] Wenzel A, Grimm C, Samardzija M, Remé CE (2005). Molecular mechanisms of light-induced photoreceptor apoptosis and neuroprotection for retinal degeneration.. Prog Retin Eye Res.

[9] Thanos C, Emerich D (2005). Delivery of neurotrophic factors and therapeutic proteins for retinal diseases.. Expert Opin Biol Ther.

[10] Andrieu-Soler C, Aubert-Pouëssel A, Doat M (2005). Intravitreous injection of PLGA microspheres encapsulating GDNF promotes the survival of photoreceptors in the rd1/rd1 mouse.. Mol Vis.

[11] Buch PK, MacLaren RE, Durán Y, Balaggan KS, MacNeil A, Schlichtenbrede FC, Smith AJ, Ali RR (2006). In contrast to AAV-mediated Cntf expression, AAV-mediated Gdnf expression enhances gene replacement therapy in rodent models of retinal degeneration.. Mol Ther.

[12] LaVail MM, Yasumura D, Matthes MT, Lau-Villacorta C, Unoki K, Sung CH, Steinberg RH (1998). Protection of mouse photoreceptors by survival factors in retinal degenerations. Invest Ophthalmol Vis Sci.

[13] Thanos CG, Bell R, O’Rourke P, Kauper K, Sherman S, Stabila P, Tao W (2004). Sustained secretion of ciliary neurotrophic factor to the vitreous using the encapsulated cell therapy-based NT-501 intraocular device.. Tissue Eng.

[14] Machida S, Chaudhry P, Shinohara T, Singh DP, Reddy VN, Chylack LT, Sieving PA, Bush RA (2001). Lens epithelium-derived growth factor promotes photoreceptor survival in light-damaged and RCS rats.. Invest Ophthalmol Vis Sci.

[15] Cayouette M, Smith SB, Becerra SP, Gravel C (1999). Pigment epithelium-derived factor delays the death of photoreceptors in mouse models of inherited retinal degenerations.. Neurobiol Dis.

[16] Léveillard T, Mohand-Saïd S, Lorentz O, Hicks D, Fintz AC, Clérin E, Simonutti M, Forster V, Cavusoglu N, Chalmel F, Dollé P, Poch O, Lambrou G, Sahel JA (2004). Identification and characterization of rod-derived cone viability factor.. Nat Genet.

[17] Sieving PA, Caruso RC, Tao W, Coleman HR, Thompson DJ, Fullmer KR, Bush RA (2006). Ciliary neurotrophic factor (CNTF) for human retinal degeneration: phase I trial of CNTF delivered by encapsulated cell intraocular implants.. Proc Natl Acad Sci USA.

[18] Emerich DF, Thanos CG (2008). NT-501: an ophthalmic implant of polymer-encapsulated ciliary neurotrophic factor-producing cells.. Curr Opin Mol Ther.

[19] WoldeMussie E, Yoles E, Schwartz M, Ruiz G, Wheeler LA (2002). Neuroprotective effect of memantine in different retinal injury models in rats.. J Glaucoma.

[20] Greenfield DS, Girkin C, Kwon YH (2005). Memantine and progressive glaucoma.. J Glaucoma.

[21] Kastin AJ, Akerstrom V, Pan W (2003). Glial cell line-derived neurotrophic factor does not enter normal mouse brain.. Neurosci Lett.

[22] Ward MS, Khoobehi A, Lavik EB, Langer R, Young MJ (2007). Neuroprotection of retinal ganglion cells in DBA/2J mice with GDNF-loaded biodegradable microspheres.. J Pharm Sci.

[23] Jiang C, Moore MJ, Zhang X, Klassen H, Langer R, Young M (2007). Intravitreal injections of GDNF-loaded biodegradable microspheres are neuroprotective in a rat model of glaucoma.. Mol Vis.

[24] Bok D, Yasumura D, Matthes M, Ruiz A, Duncan JL, Chappelow AV, Zolutukhin S, Hauswirth W, LaVail MM (2002). Effects of adeno-associated virus-vectored ciliary neurotrophic factor on retinal structure and function in mice with a P216L rds/peripherin mutation.. Exp Eye Res.

[25] Geroski DH, Edelhauser HF (2001). Transscleral drug delivery for posterior segment disease.. Adv Drug Deliv Rev.

[26] Lee SB, Geroski DH, Prausnitz MR, Edelhauser HF (2004). Drug delivery through the sclera: effects of thickness, hydration, and sustained release systems.. Exp Eye Res.

[27] Lawrence JM, Keegan DJ, Muir EM, Coffey PJ, Rogers JH, Wilby MJ, Fawcett JW, Lund RD (2004). Transplantation of Schwann cell line clones secreting GDNF or BDNF into the retinas of dystrophic Royal College of Surgeons rats.. Invest Ophthalmol Vis Sci.

[28] Gamm DM, Wang S, Lu B, Girman S, Holmes T, Bischoff N, Shearer RL, Sauvé Y, Capowski E (2007). Protection of visual functions by human neural progenitors in a rat model of retinal disease.. PLoS One.

[29] MacDonald IM, Sauvé Y, Sieving PA (2007). Preventing blindness in retinal disease: ciliary neurotrophic factor intraocular implants.. Can J Ophthalmol.

[30] Yaneza M, Gilthorpe JD, Lumsden A, Tucker AS (2002). No evidence for ventrally migrating neural tube cells from the mid- and hindbrain.. Dev Dyn.

[31] Adams DJ, Biggs PJ, Cox T, Davies R, van der Weyden L, Jonkers J, Smith J, Plumb B, Taylor R, Nishijima I, Yu Y, Rogers J, Bradley A (2004). Mutagenic insertion and chromosome engineering resource (MICER).. Nat Genet.

[32] Hooper M, Hardy K, Handyside A, Hunter S, Monk M (1987). HPRT-deficient (Lesch-Nyhan) mouse embryos derived from germline colonization by cultured cells.. Nature.

[33] Bibb LC, Holt JK, Tarttelin EE, Hodges MD, Gregory-Evans K, Rutherford A, Lucas RJ, Sowden JC, Gregory-Evans CY (2001). Temporal and spatial expression patterns of the CRX transcription factor and its downstream targets. Critical differences during human and mouse eye development. Hum Mol Genet.

[34] Frasson M, Picaud S, Léveillard T, Simonutti M, Mohand-Said S, Dreyfus H, Hicks D, Sabel J (1999). Glial cell line-derived neurotrophic factor induces histologic and functional protection of rod photoreceptors in the rd/rd mouse. Invest Ophthalmol Vis Sci.

[35] Steinberg RH, Flannery JG, Naash M, Oh P, Matthes MT, Yasumura D, Lau-Villacorta C, Chen J, LaVail MM. Transgenic rat models of inherited retinal degeneration caused by mutant opsin genes. ARVO Annual Meeting; 1996 April 21–26; Fort Lauderdale (FL).

[36] McGee-Sanftner LH, Abel H, Hauswirth WW, Flannery JG (2001). Glial cell line derived neurotrophic factor delays photoreceptor degeneration in a transgenic rat model of retinitis pigmentosa.. Mol Ther.

[37] Chen J, Makino CL, Peachey NS, Baylor DA, Simon MI (1995). Mechanisms of rhodopsin inactivation in vivo as revealed by a COOH-terminal truncation mutant.. Science.

[38] Berson EL, Rosner B, Weigel-DiFranco C, Dryja TP, Sandberg MA (2002). Disease progression in patients with dominant retinitis pigmentosa and rhodopsin mutations.. Invest Ophthalmol Vis Sci.

[39] Guerin K, Gregory-Evans CY, Hodges MD, Moosajee M, Mackay DS, Gregory-Evans K, Flannery JG (2008). Systemic aminoglycoside treatment in rodent models of retinitis pigmentosa.. Exp Eye Res.

[40] Nordhoff V, Hübner K, Bauer A, Orlova I, Malapetsa A, Schöler HR (2001). Comparative analysis of human, bovine, and mouse Oct-4 upstream promoter sequences.. Mamm Genome.

[41] Otani A, Dorrell MI, Kinder K, Moreno SK, Nusinowitz S, Banin E, Heckenlively J, Friedlander M (2004). Rescue of retinal degeneration by intravitreally injected adult bone marrow-derived lineage-negative hematopoietic stem cells.. J Clin Invest.

[42] Canola K, Angénieux B, Tekaya M, Quiambao A, Naash MI, Munier FL, Schorderet DF, Arsenijevic Y (2007). Retinal stem cells transplanted into models of late stages of retinitis pigmentosa preferentially adopt a glial or a retinal ganglion cell fate.. Invest Ophthalmol Vis Sci.

[43] Meyer JS, Katz ML, Maruniak JA, Kirk MD (2006). Embryonic stem cell-derived neural progenitors incorporate into degenerating retina and enhance the survival of host photoreceptors.. Stem Cells.

[44] Lau D, McGee LH, Zhou S, Rendahl KG, Manning WC, Escobedo JA, Flannery JG (2000). Retinal degeneration is slowed in transgenic rats by AAV-mediated delivery of FGF-2.. Invest Ophthalmol Vis Sci.

[45] Lin LF, Doherty DH, Lile JD, Bektesh S (1993). Collins F. GDNF: a glial cell line-derived neurotrophic factor for midbrain dopaminergic neurons.. Science.

[46] Akerud P, Canals JM, Snyder EY, Arenas E (2001). Neuroprotection through delivery of glial cell line-derived neurotrophic factor by neural stem cells in a mouse model of Parkinson's disease.. J Neurosci.

[47] Perez-Navarro E, Arenas E, Marco S, Alberch J (1999). Intrastriatal grafting of a GDNF-producing cell line protects striatonigral neurons from quinolinic acid excitotoxicity in vivo.. Eur J Neurosci.

[48] Jing S, Wen D, Yu Y, Holst PL, Luo Y, Fang M, Tamir R, Antonio L, Hu Z, Cupples R, Louis JC, Hu S, Altrock BW, Fox GM (1996). GDNF-induced activation of the ret protein tyrosine kinase is mediated by GDNFR-alpha, a novel receptor for GDNF.. Cell.

[49] Nosrat CA, Tomac A, Lindqvist E, Lindskog S, Humpel C, Strömberg I, Ebendal T, Hoffer BJ, Olson L (1996). Cellular expression of GDNF mRNA suggests multiple functions inside and outside the nervous system.. Cell Tissue Res.

[50] Pachnis V, Mankoo B, Costantini F (1993). Expression of the c-ret proto-oncogene during mouse embryogenesis.. Development.

[51] Hauck SM, Kinkl N, Deeg CA, Swiatek-de Lange M, Schöffmann S, Ueffing M (2006). GDNF family ligands trigger indirect neuroprotective signaling in retinal glial cells.. Mol Cell Biol.

[52] Müller F-J, Snyder EY, Loring JF (2006). Gene therapy: can neural stem cells deliver?. Nat Rev Neurosci.

[53] Ourednik J, Ourednik V, Lynch WP, Schachner M, Snyder EY (2002). Neural stem cells display an inherent mechanism for rescuing dysfunctional neurons.. Nat Biotechnol.

[54] Beider K, Abraham M, Peled A (2008). Chemokines and chemokine receptors in stem cell circulation.. Front Biosci.

[55] Fox JM, Chamberlain G, Ashton BA, Middleton J (2007). Recent advances in understanding mesenchymal stem cell trafficking.. Br J Haematol.

[56] Repetto R, Stocchino A, Cafferata C (2005). Experimental investigation of vitreous humour motion with a human eye model.. Phys Med Biol.

[57] Stocchino A, Repetto R, Cafferata C (2007). Eye rotation induced dynamics of a Newtonian fluid within the vitreous cavity: the effect of chamber shape.. Phys Med Biol.

[58] Fändrich F, Dresske B, Bader M, Schulze M (2002). Embryonic stem cells share immune-privileged features relevant for tolerance induction.. J Mol Med.

[59] Swijnenburg RJ, Schrepfer S, Cao F, Pearl JI, Xie X, Connolly AJ, Robbins RC, Wu JC (2008). In vivo imaging of embryonic stem cells reveals patterns of survival and immune rejection following transplantation.. Stem Cells Dev.

[60] McGill TJ, Lund RD, Douglas RM, Wang S, Lu B, Silver BD, Secretan MR, Arthur JN, Prusky GT (2007). Syngeneic Schwann cell transplantation preserves vision in RCS rat without immunosuppression.. Invest Ophthalmol Vis Sci.

[61] Dyer MA, Cepko CL (2001). p27Kip1 and p57Kip2 regulate proliferation in distinct retinal progenitor cell populations.. J Neurosci.

[62] Nutt JG, Burchiel KJ, Comella CL, Jankovic J, Lang AE, Laws ER, Lozano AM, Penn RD, Simpson RK, Stacy M, Wooten GF, ICV GDNF Study Group. (2003). (Implanted intracerebroventricular-glial cell line-derived neurotrophic factor). Randomized, double-blind trial of glial cell line-derived neurotrophic factor (GDNF) in PD.. Neurology.

[63] Hovland DN, Boyd RB, Butt MT, Engelhardt JA, Moxness MS, Ma MH, Emery MG, Ernst NB, Reed RP, Zeller JR, Gash DM, Masterman DM, Potter BM, Cosenza ME, Lightfoot RM (2007). Six-month continuous intraputamenal infusion toxicity study of recombinant methionyl human glial cell line-derived neurotrophic factor (r-metHuGDNF in rhesus monkeys.. Toxicol Pathol.

[64] Gash DM, Zhang Z, Cass WA, Ovadia A, Simmerman L, Martin D, Russell D, Collins F, Hoffer BJ, Gerhardt GA (1995). Morphological and functional effects of intranigrally administered GDNF in normal rhesus monkeys.. J Comp Neurol.

[65] Vinores SA, Küchle M, Derevjanik NL, Henderer JD, Mahlow J, Green WR, Campochiaro PA (1995). Blood-retinal barrier breakdown in retinitis pigmentosa: light and electron microscopic immunolocalization.. Histol Histopathol.

[66] Nishikiori N, Osanai M, Chiba H, Kojima T, Mitamura Y, Ohguro H, Sawada N (2007). Glial cell-derived cytokines attenuate the breakdown of vascular integrity in diabetic retinopathy.. Diabetes.

[67] Crook JM, Peura TT, Kravets L, Bosman AG, Buzzard JJ, Horne R, Hentze H, Dunn NR, Zweigerdt R, Chua F, Upshall A, Colman A (2007). The generation of six clinical-grade human embryonic stem cell lines.. Cell Stem Cell.

[68] Unger C, Skottman H, Blomberg P, Dilber MS, Hovatta O (2008). Good manufacturing practice and clinical-grade human embryonic stem cell lines.. Hum Mol Genet.

